# Predictive value of neuron-specific enolase following moderate and severe traumatic brain injury: a systematic review and meta-analysis

**DOI:** 10.1186/cc10918

**Published:** 2012-03-20

**Authors:** E Mercier, AF Turgeon, A Boutin, F Lauzier, R Zarychanski, P Archambault, J Granton, F Lamontagne, L Moore, F Rousseau, F Légaré, E Randell, J Lacroix, J Lapointe, D Fergusson

**Affiliations:** 1 Université Laval, Québec, Canada; 2 University of Manitoba, Canada; 3 Université of Toronto, Ontario, Canada; 4 Université de Sherbrooke, Québec, Canada; 5 Memorial University, NewFoundland, Canada; 6 Université de Montréal, Québec, Canada; 7 Ottawa Hospital Research Institute, Ontario, Canada

## Introduction

Biomarkers such as the neuron-specific enolase (NSE) have been proposed as potential prognostic markers following traumatic brain injury (TBI) [1,2]. However, the use of NSE is not currently recommended for prognostic evaluation. Our objective was to systematically review the prognostic value of NSE levels following moderate or severe TBI.

## Methods

We systematically searched MEDLINE, Embase, Cochrane, Biosis, Scopus, Trip, references of eligible studies, reviews and conference proceedings. Eligible studies were cohort studies including ≥4 patients with moderate or severe TBI having measured the association between NSE levels (first 24 hours) and mortality or the Glasgow Outcome Scale (GOS). Independently, two reviewers selected studies and extracted data using a standardized form. Pooled results using random-effect models were used using weighted mean differences (WMD); heterogeneity was assessed using I^2 ^tests. Sensitivity analyses were planned to explain statistical heterogeneity (for example, extracerebral injuries).

## Results

We retrieved 4,711 citations and included 22 studies (*n *= 757). Seventeen studies used the GOS as an outcome measure while 10 studies reported mortality. Most studies evaluated outcomes at 6 months or beyond (range: 1 to 12 months). Ten studies could not be included in the pooled analyses: three reported mean levels of serial samplings, two presented peak levels, two reported medians, one did not report any measure of dispersion and data could not be extracted from two studies. We observed a significant association between serum NSE levels and mortality (five studies: WMD 25.90 (95% CI 15.97 to 35.83), I^2 ^= 60%) and GOS ≤3 (10 studies: WMD 17.69 (95% CI 12.14 to 23.24), I^2 ^= 64%). Similar results were found with or without extracerebral injuries. The number of studies included in pooled analyses precluded performing relevant sensitivity analyses.

## Conclusion

We observed a significant association between serum NSE levels and unfavorable outcomes (mortality or GOS ≤3) not influenced by extracerebral injuries. Further studies need to evaluate the usefulness of serum NSE levels for prognosis assessment in TBI and its potential impact on clinical decision-making.
